# Acute intraocular pressure responses changes during dynamic resistance training in primary open-angle glaucoma patients and age-matched controls

**DOI:** 10.1007/s00417-025-06814-9

**Published:** 2025-04-17

**Authors:** María Dolores Morenas-Aguilar, Cristina González-Hernández, Daniel Marcos-Frutos, Sergio Miras-Moreno, María José López-Gómez, Amador García-Ramos, Jesús Vera

**Affiliations:** 1 Department of Physical Education and Sport, Faculty of Sport Sciences, University of Granada, Granada, Spain; 2 Department of Ophthalmology, Virgen de Las Nieves University Hospital, Granada, Spain; 3 Department of Sports Sciences and Physical Conditioning, Faculty of Education, Universidad Católica de La Santísima Concepción, Concepción, Chile; 4 CLARO (Clinical and Laboratory Applications of Research in Optometry) Laboratory, Department of Optics, Faculty of Sciences, University of Granada, Granada, Spain; 5 New England College of Optometry, Boston, MA USA

**Keywords:** Leg extension, Ocular health, Biceps curl, Physical activity, Glaucoma management

## Abstract

**Background:**

Physical exercise has been proposed as a feasible strategy for preventing and managing glaucoma by modulating intraocular pressure (IOP) and ocular perfusion pressure (OPP). The primary objective of this cross-sectional study was to assess the IOP and OPP responses to dynamic resistance exercises (leg extension and biceps curl).

**Methods:**

Twenty-six patients with primary open-angle glaucoma (POAG) (age = 68.9 ± 8.1 years) and 18 healthy age-matched controls (age = 69.6 ± 5.9 years) were recruited. Participants performed one set of 10 repetitions of both exercises at low- (light bar) and moderate-intensity (15RM). IOP and blood pressure were measured at baseline and after 1 and 5 min of passive recovery. Additionally, IOP was measured during training after each of the 10 repetitions.

**Results:**

Our data showed a progressive IOP increase throughout the sets of leg extension and biceps curl exercises when performed at moderate intensity (*p* < 0.001). Remarkably, POAG patients showed a smaller IOP increase compared to controls (*p* = 0.048). The between-group differences for IOP changes were higher during the 10 exercise repetitions at moderate-intensity for both leg extension (average IOP rise: POAG = 0.3 ± 0.6 mmHg vs. control = 2.3 ± 0.7 mmHg) and biceps curl (average IOP rise: POAG = 1.4 ± 0.6 mmHg vs. control = 3.4 ± 0.8 mmHg) exercises. No changes in OPP were observed.

**Conclusions:**

The findings of this study suggest that moderate-intensity dynamic resistance training is a safe intervention for potentially improving physical fitness in medically treated POAG patients.

**Supplementary Information:**

The online version contains supplementary material available at 10.1007/s00417-025-06814-9.

## Abstract (highlights)

**Key messages:**

*****What is known***:**

- Although physical exercise has been proposed as a strategy for glaucoma prevention and management, resistance exercises, especially when performed at high intensity, lead to an acute increase in intraocular pressure.

*****What is new***:**

- The intraocular pressure increases during low- to moderate-intensity dynamic sets of leg extension and biceps curl in POAG patients is clinically irrelevant, meanwhile ocular perfusion pressure remains stable.
- The better intraocular pressure response to dynamic strength exercises in POAG patients in comparison with controls could be associated with the effectiveness of the eye drop treatment (prostaglandin analogues or a combination of prostaglandin analogues and beta-blockers).
- Moderate-intensity dynamic resistance training appears to be a safe strategy and should be considered by trainers and eye care specialists working with POAG patients on eye drop treatment.

**Supplementary Information:**

The online version contains supplementary material available at 10.1007/s00417-025-06814-9.

## Introduction

Resistance training provides a range of performance and health benefits for both athletes and clinical populations. Athletes typically engage in resistance training to enhance performance or prevent injuries, while healthy adults and those with medical conditions use it to improve functional capacity [1]. Regular resistance training has been shown to effectively manage and prevent various chronic conditions (e.g., arthritis, diabetes, and heart disease) by slowing disease progression and enhancing quality of life through the maintenance of muscle quality [2, 3]. Additionally, resistance training reduces the risk of fall-related injuries and neurodegenerative disorders, while also promoting bone, cardiovascular, and mental health [4]. Resistance training may help reduce the risk of frailty commonly observed in older adults with hypertension, glaucoma, or diabetes. Additionally, it may slow physical and cognitive decline, reduce disability, and lower mortality risk [5]. However, an individualized exercise prescription is essential, tailored to the patient's condition and underlying medical issues.

Concerns about eye health, particularly regarding glaucoma, the leading cause of irreversible blindness worldwide, have grown significantly in recent years, especially in relation to lifestyle habits [6]. The only proven strategy for managing glaucoma is lowering intraocular pressure (IOP), with first-line interventions including eye drops and laser trabeculoplasty, alongside supportive lifestyle factors such as physical exercise, caffeine intake, and psychological habits [7]. In fact, regular physical exercise has been shown to positively affect ocular health, reducing the risk of various eye diseases [8]. Observational studies have reported lower IOP and higher ocular perfusion pressure (OPP) values in individuals with greater fitness levels [8–10]. Specifically, glaucoma patients using topical medication experience a greater IOP-lowering effect after one month of daily exercise compared to inactive patients [11].

Overall, physical exercise has been proposed as a feasible strategy for preventing and managing glaucoma alongside medical treatment [12]. Among the various factors influencing the IOP responsiveness to physical exercise, the type of exercise performed is particularly relevant [13]. While low to moderate intensity endurance exercises tend to reduce IOP [14, 15], resistance exercises, particularly when executed with high levels of effort (e.g., heavy loads and close proximity to failure) cause an acute rise in IOP [6, 13, 16]. This increase is further exacerbated by breath-holding and the Valsalva maneuver during high-intensity resistance exercises [17]. Additionally, exercises involving larger muscle groups (e.g., squats) result in greater IOP changes than those targeting smaller muscle groups (e.g., biceps curls) [1, 6]. Of the few studies comparing upper- and lower-body exercises, two found squats produced greater IOP fluctuations compared to biceps curl and military press [6, 18]. To date, most studies have been conducted on healthy individuals, but glaucoma patients, who have an impaired outflow facility [19, 20], may experience different IOP responses [21, 22]. Further studies are needed to explore IOP changes during resistance exercises in glaucoma patients.

Elevated IOP is the primary risk factor for glaucoma, but vascular factors also play a crucial role in its pathogenesis [23]. Higher OPP has been associated with a lower incidence of glaucoma and slower disease progression [10]. While endurance and resistance exercises generally increase OPP [12], resistance exercise sets performed to failure have demonstrated to reduce OPP in healthy individuals [18]. The relationship between physical exercise and OPP is complex, influenced by the autoregulatory mechanisms that regulate blood flow [24]. In glaucoma patients, the altered outflow facility may contribute to differences in blood flow regulation during certain resistance exercises compared to healthy individuals [19]. Although few studies have investigated blood flow responses to isometric exercise in primary open-angle glaucoma (POAG) patients [19, 25, 26], dynamic resistance exercises remain unexplored. Thus, further research on resistance exercise prescriptions to improve OPP in POAG patients with impaired outflow resistance is warranted.

The primary objective of this study was to assess the IOP and OPP responses to dynamic lower- and upper-body resistance exercises. To ensure participant safety and proper technical execution while minimizing the risk of injury, we selected monoarticular exercises, such as leg extensions and biceps curls, instead of compound exercises like squats. Additionally, the secondary objectives included: (i) comparing IOP and OPP responses between POAG patients and age-matched controls, (ii) comparing IOP and OPP responses between lower- and upper-body exercises (leg extension vs. biceps curl) and (iii) comparing IOP and OPP responses when the exercises are performed at low and moderate intensities. We hypothesized that moderate-intensity dynamic resistance exercise would result in a moderate increase in IOP [1]. While the results of the comparison between the POAG and control groups are uncertain due to the lack of related studies, however, the altered mechanism of regulation of aqueous humor in POAG patients may lead to a greater increase compared to control individuals [22].

## Methods

### Participants

The required sample size was determined by an a-priori power analysis using the G*Power 3.1 software [27], considering an analysis of variance (ANOVA) with within- and between-participant factors. For this analysis, we have considered an effect size (Cohen´s f) of 0.2, which corresponds to approximately 40% of the magnitude of the change found for IOP levels during aerobic exercise in POAG patients [28], an α-level of 0.05 and a power of 0.80. This calculation projected that the required number of participants to ensure the detection of significant differences was 36 subjects (18 POAG and 18 controls). For this study, 26 POAG patients (age = 68.9 ± 8.1 years, body mass = 71.9 ± 12.2 kg, height = 165.7 ± 10.4 cm) and 18 healthy older adults (age = 69.6 ± 5.9 years, body mass = 73.3 ± 12.9 kg, height = 162.9 ± 9.4 cm) were recruited. The POAG group was formed by 13 men and 13 women recruited from the Unit of Glaucoma at Virgen de las Nieves University Hospital (Granada, Spain), whereas the control group was formed by 6 men and 12 women. All participants were of Caucasian race. For POAG patients’ recruitment, the disease diagnosis was based on objective criteria such as glaucomatous optic nerve head changes and visual field defects consistent with glaucoma, and all patients were medically treated with prostaglandin analogues or a combination of prostaglandin analogue and beta-blockers. In addition, the inclusion criteria for all participants were as follows: (a) have a sufficient level of mobility to perform low to moderate intensity physical activity; (b) be free of other underlying diseases that could be negatively affected by exercise; (c) absence of any surgical intervention for glaucoma treatment; and (d) be between 60 and 80 years old. Participants were instructed to avoid caffeine, alcohol, and strenuous exercise before testing. This study was conducted in accordance with the Code of Ethics of the World Medical Association (Declaration of Helsinki) and was approved by the Institutional Review Board (IRB approval December 22, 2022).

### Study design

A cross-sectional design was used to investigate the influence of dynamic biceps curl and leg extension exercises on IOP behaviour at low and moderate intensities in POAG patients and sex-matched controls. Participants attended two days to the laboratory, with both sessions being separated by at least 48 h. In order to avoid circadian fluctuations, participants were tested at the same time of the day (± 2 h). In the first session, the 15 repetitions maximum (RM) load in the biceps curl and leg extension exercises was determined. In the second session, participants randomly performed 1 set of 10 repetitions of each exercise at low intensity (0.5 kg bar for biceps curl and 5 kg for leg extension) and moderate intensity (15RM). IOP was measured at baseline (upon arrival), after each of the 10 repetitions, and after 1 and 5 min of passive recovery. Additionally, blood pressure was measured at baseline, immediately after exercise cessation, and after 5 min of passive recovery. All participants were tested under similar environmental conditions (approximately 22 °C and 60% humidity) and were not allowed to drink or eat during the assessment.

### Procedures

**Fig. 1:**
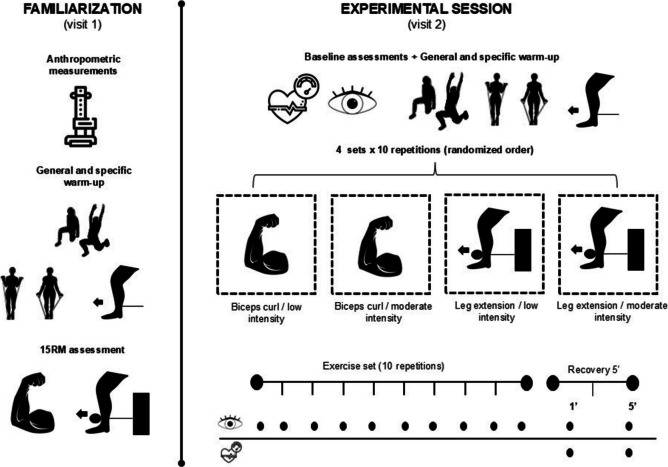
A schematic illustration of the study procedures in both experimental sessions

A schematic overview of the study protocol is depicted in Fig. 1. The first session (familiarization session) was used to determine the 15RM (i.e., the load at which participants can perform a maximum of 15 repetitions) for the biceps curl (free weights) and leg extension (leg extension machine) exercises. At the beginning of the session, participants' age, body mass, and height were obtained. After that, participants performed a general warm-up of full-body mobility and practiced the exercises with elastic bands, a light bar and the leg extension machine. The 15RM load for biceps curls and leg extension was determined through an incremental loading test. The weight in the sets of each exercise was progressively incremented until the 15RM load was reached. The set was stopped when an experienced strength and conditioning coach identified that participants could perform more than 15 repetitions. Three minutes of passive rest were implemented between sets. The order of the exercises was randomized.

In the second session (experimental session), IOP was assessed at baseline and then participants conducted the same warm-up as the described for the first session. After that, participants performed one set of 10 repetitions against the 15RM load and one set of 10 repetitions with a light bar (0.5 kg) for biceps curl and leg extension with 10 min of rest between exercises. The IOP was measured immediately after each repetition at the resting position to avoid the influence of active effort as Vera et al. previously reported [6]. Participants were asked to maintain a normal breathing pattern during both exercises.

### Measurement equipment

IOP was measured using a portable rebound tonometer (Icare IC200, Tiolat Oy, Helsinki, Finland). Participants were instructed to fixate on a distant target while six consecutive measurements were taken against the central cornea. The average of these six measurements was used for further analysis. Blood pressure was measured using a wrist digital automatic monitor (RS6 HEM-6221, Omron, Kyoto, Japan), which has been validated for blood pressure assessment [29]. Ocular perfusion pressure (OPP) was estimated from the assessment of IOP and blood pressure (OPP = [95/140 × mean arterial pressure] – IOP).

### Statistical analysis

Descriptive data are presented as means ± standard deviation. The normal distribution of the data was checked with the Shapiro–Wilk test, and the homogeneity of variances with the Levene’s test (*p* > 0.05). For the analysis of IOP changes, a repeated measures analysis of variance was carried out with the *intensity* (low, moderate), *exercise* (biceps curl, leg extension) and *point of measure* (baseline, after each repetition, and after 1 and 5 min of passive recovery) as the within-participant factors, whereas the *group* (POAG, control) was considered as the only between-participant factor. For the analysis of OPP changes, data were submitted to another repeated measures analysis of variance with the *intensity* (low, moderate), *exercise* (biceps curl, leg extension) and *point of measure* (baseline, immediately after exercise cessation and after 5 min of passive recovery) as the within-participant factors, and the *group* (POAG, control) as the only between-participant factor. The Holm–Bonferroni procedure was applied when performing pairwise comparisons. Statistical significance was set at *p* ≤ 0.05. The magnitude of the differences was reported by the by the Cohen’s d effect size (d) and partial eta squared (ƞ2p) for T and F tests, respectively. All statistical analyses were performed using the JASP statistics package (version 0.18.3).

## Results

First, we checked the between-group differences for baseline IOP and OPP assessments and fitness level (i.e., biceps curl and leg extension 15RM). There were not statistically significance differences for baseline IOP (POAG = 17.3 ± 4.4 mmHg, control = 16.6 ± 3.2 mmHg; *t* = −0.625, *p* = 0.535, *d* = 0.19), OPP (POAG = 53.8 ± 9.8 mmHg, control = 49.9 ± 8.0 mmHg; t = −1.41, *p* = 0.165, d = 0.43), biceps curl 15RM (POAG = 11.7 ± 4.6 kg control = 10.1 ± 2.8 kg; *t* = −1.290, *p* = 0.204, *d* = 0.395) and leg extension 15RM (POAG = 21.1 ± 12.9 kg, control = 21.7 ± 11.9 kg; *t* = 0.157, *p* = 0.876, *d* = 0.048) between the POAG and control groups.

**Fig. 2:**
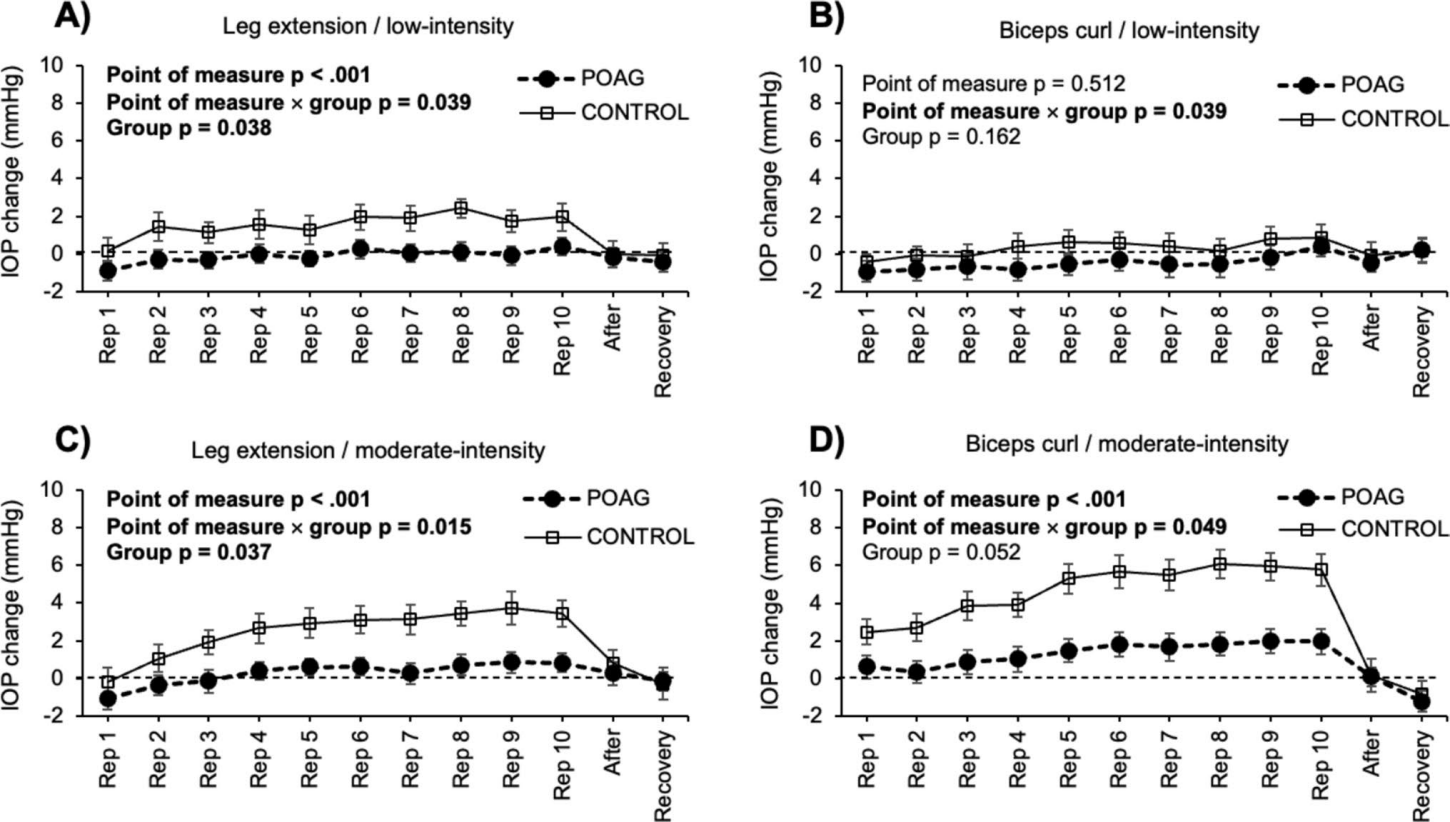
Effects of leg extension and biceps curl exercises at two intensities on intraocular pressure in the primary open-angle glaucoma (POAG) and control groups. The specific experimental condition is depicted above each panel (panel **A** = leg extension exercise at low-intensity, panel **B** = biceps curl exercise at low intensity, panel **C** = leg extension exercise at moderate intensity, and panel **D** = biceps curl exercise at moderate intensity). Error bars represent the 95% confidence intervals. Bold font has been used to highlight statistically significant effects (*p* < 0.05). IOP = intraocular pressure; Rep = repetition; After = measurement taken after completing the set (within 1 min); Recovery = measurement taken after 5 min of completing the set

The 4-way ANOVA applied for the IOP changes revealed statistical significance for the main effects of *intensity* (F = 49.1, *p* < 0.001, ƞ^2^_p_ = 0.54), *point of measure* (F = 23.3, *p* < 0.001, ƞ^2^_p_ = 0.36), and *group* (F = 4.2, *p* = 0.048, ƞ^2^_p_ = 0.09). However, the main effect of *exercise* did not reach statistical significance (F = 0.9, *p* = 0.339, ƞ^2^_p_ = 0.02). The interaction effects *point of measure* × *group* (F = 4.9, *p* < 0.001, ƞ^2^_p_ = 0.11), *exercise* × *intensity* (F = 39.8, *p* < 0.001, ƞ^2^_p_ = 0.49), *intensity* × *point of measure* (F = 10.8, *p* < 0.001, ƞ^2^_p_ = 0.20), and *exercise* × *intensity* × *point of measure* (F = 4.7, *p* < 0.001, ƞ^2^_p_ = 0.10) showed statistical significance, whereas the rest of interactions did not reach statistically significant differences (*p* > 0.184 in all cases). Post-hoc analyses revealed higher IOP increments in the moderate- in comparison to the low-intensity condition (corrected *p*-value < 0.001, Cohen’s *d* = 0.38), and POAG patients experienced a lower IOP increase when compared with the control group (corrected *p*-value = 0.048, Cohen’s *d* = 0.52). Also, there was a linear IOP rise during exercise execution, with IOP returning to baseline levels immediately after completing the exercise (Fig. 2).

Complementarily, four separate ANOVAs for both exercises (leg extension and biceps curl) and intensities (low and moderate) were carried out to explore the differences between the POAG and control groups (see Fig. 2). For the biceps curl exercise, POAG patients tended towards showing a more stable IOP response than controls in the moderate-intensity condition (F = 4.0, *p* = 0.052, ƞ^2^_p_ = 0.09), but the differences in the low-intensity condition were statistically and clinically insignificant (F = 2.0, *p* = 0.162, ƞ^2^_p_ = 0.05). When considering the leg extension exercise, controls showed greater IOP increments than POAG patients during the execution of the 10 exercise repetitions in both exercise intensities (low-intensity: F = 4.6, *p* = 0.038, ƞ^2^_p_ = 0.10; and moderate-intensity: F = 4.6, *p* = 0.037, ƞ^2^_p_ = 0.10). However, these differences were only evident during the execution of the training set, with IOP levels rapidly returning to baseline levels after exercise cessation (see Fig. 2).

Regarding OPP, there were not statistically significant differences for the main effects of *exercise* (F = 0.6, *p* = 0.431), *intensity* (F = 2.1, *p* = 0.152), *point of measure* (F = 0.2, *p* = 0.700), and *group* (F = 0.1, *p* = 0.722). Also, all interactions were far from reaching statistical significance (all *p*-values > 0.195).

## Discussion

This study aimed to assess the IOP and OPP responses to dynamic resistance exercise (leg extension and biceps curl) in POAG patients and age-matched controls. We found a progressive IOP increase throughout the sets of leg extension and biceps curl exercises when performed at moderate intensity. However, IOP returned to baseline levels immediately after exercise cessation. Remarkably, POAG patients showed a smaller IOP increase compared to the control group. The magnitude of these differences between groups was higher in the more physically demanding conditions, observing a heightened IOP increment for the control group in comparison to the POAG patients during the 10 exercise repetitions in the moderate-intensity conditions of the leg extension (POAG = 0.3 ± 0.6 mmHg vs. control = 2.3 ± 0.7 mmHg) and biceps curl (POAG = 1.4 ± 0.6 mmHg vs. control = 3.4 ± 0.8 mmHg) exercises. No changes in OPP were observed across any exercise or intensity in either group. Taken together, this study supports the use of low- to moderate-intensity leg extension and biceps curl exercises as a safe training strategy for POAG patients undergoing eye drops treatment.

In line with previous studies, we found an acute increase in IOP levels during the execution of dynamic resistance exercises [1]. Although repeated IOP increases could potentially harm retinal function, the rise observed in this study was clinically modest, reaching 0.8 ± 0.6 mmHg for POAG patients and 2.8 ± 0.7 mmHg for the control group at moderate intensity and, −0.3 ± 0.4 mmHg for POAG patients and 1.2 ± 0.3 mmHg for the control group at low intensity. However, other studies have found a greater IOP elevation (up to 28 mmHg) during dynamic and isometric resistance exercises [1, 30]. This may be explained by the different physiological responses associated with the exercise intensity (i.e., breathing pattern). In fact, performing high intensity resistance exercises (i.e., near to failure) tends to alter the normal breathing pattern, leading to the Valsalva manoeuvre or breath-holding strategies, which can exacerbate IOP changes [18, 31]. In contrast, low- to moderate-intensity exercises tend to promote a steady breathing pattern, allowing to maintain an stable IOP behaviour [30]. In the present study, participants were advised to avoid the Valsalva manoeuvre; however, it was not objectively monitored, and the possibility of unintentional breath-holding cannot be ruled out. Nonetheless, we hypothesized that performing leg extensions and biceps curls at higher intensities would require greater physical effort, resulting in a more pronounced increase in IOP. Therefore, exercise intensity and the associated breathing pattern should be carefully considered by trainers and eye care specialists in the prevention and management of glaucoma. While high-intensity resistance exercises result in significant IOP increments [18, 32], moderate-intensity exercise appear to be effective for improving fitness level without compromising eye health. Future studies using the biceps curl and leg extension exercises at higher intensities should be performed to explore its impact on IOP levels.

Additionally, previous results have shown that accumulated repetitions lead to greater IOP levels (corrected *p*-value < 0.001, Cohen’s *d* = 0.43) [6]. Beyond exercise intensity, longer set durations increase effort, resulting in a pronounced impact on IOP [1]. To minimize undesirable IOP alterations, performing only half of the maximum possible repetitions (medium level of effort) should be recommended for POAG patients or those at risk. In the present study, a brief rest period (1 min) was sufficient for IOP to return to baseline levels. Recent research has also indicated that even significant IOP elevations from resistance training are transient, with recovery occurring in a short time [13]. Thus, incorporating intra-set rests may be an alternative to prevent continuous IOP elevation. Moreover, unlike previous studies comparing various resistance exercises, we did not observe significant differences between leg extension and biceps curl exercises. In general, exercises that recruit larger muscle masses (e.g., squat) lead to greater IOP increases compared to those involving smaller muscle groups (e.g., biceps curl) [1, 6, 18]. However, when comparing exercises with similar muscle mass recruitment (e.g., biceps curl vs. calf raise), upper-body exercises tend to provoke a higher IOP increase [18]. In this study, we found a greater IOP increase during biceps curl compared to leg extension at moderate intensity (2.2% for POAG patients and 9.3% for controls).

Overall, during resistance exercise in healthy adults, IOP acutely rises, with varying degrees of fluctuation [1]. In line with these findings, the present results showed an IOP elevation with low- to moderate-intensity dynamic exercises in POAG patients and age-matched controls. Contrary to our hypothesis, a lower IOP increase was found in POAG patients in comparison to the control group. Also, greater differences between groups appear in the more physically demanding conditions when IOP tends to increase sharply [18]. This result may be attributed to the effectiveness of eye drops used to regulate the altered outflow facility in this cohort, as all POAG patients in the study were treated with prostaglandin analogues or a combination of prostaglandin analogues and beta-blockers. The IOP increase in POAG patients at both intensities was minimal (< 1 mmHg), supporting the combined use of medication and physical training [30]. Moreover, although the sample size of the POAG group was relatively small, we explored the potential effect of eye drop treatment (prostaglandin analogues vs. a combination of prostaglandin analogues and beta-blockers) on the IOP response to dynamic resistance training. This analysis revealed that the IOP changes in POAG patients were independent of the eye drop treatment (*p* = 0.902). Therefore, it appears that glaucoma patients treated with eye drops could benefit from a higher training intensity without suffering from instability of IOP values.

Regarding vascular factors, physical exercise has been linked with higher OPP values [12], which may reduce the risk of glaucoma development and progression [10]. Isometric exercises have shown increased blood flow and OPP levels in glaucoma patients [19, 25]. However, we found no significant differences in OPP levels after low- to moderate-intensity dynamic resistance exercises. High-effort exercises, in contrast, have been shown to cause an acute OPP reduction [18], highlighting the complex relationship between physical exercise and OPP regulation. The medication regimen of the POAG patients in this study may have influenced their altered outflow facility response, permitting them an effective regulation of OPP levels during exercise.

This study provides new exercise strategies for managing glaucoma, showing that low- to moderate-intensity dynamic resistance exercise could be a feasible option for enhancing muscle strength in POAG patients with controlled IOP levels. However, these results should be interpreted cautiously due to the following limitations. Although leg extension and biceps curl are commonly included in resistance training programs, our findings should not be generalized to other types of exercises, as IOP and OPP responses could vary. Additionally, while participants were instructed to maintain steady breathing, breathing patterns are known to be important modulators of the IOP response to resistance exercises [30, 33]. Furthermore, the use of eye drops appears to influence the IOP response to exercise in POAG patients; therefore, other types of glaucoma or different medications may affect the functioning of the aqueous humour outflow system [22]. Thus, the external validity of these findings should be explored in other types of glaucoma or with different treatment regimens. Also, some studies have observed that IOP and OPP responses to exercise are influenced by sex and fitness level [13, 34]. Future studies with a larger sample size should further explore these relationships in the context of the current findings. Lastly, randomized clinical trials are needed to assess the long-term effects of dynamic resistance training on glaucoma management.

## Conclusion

IOP shows a slight (clinically irrelevant) increase during dynamic sets of leg extension and biceps curl exercises executed at low to moderate intensities in POAG patients. However, age-matched controls experienced a heightened IOP rise during resistance exercise. This finding could be associated with the effectiveness of the eye drop treatment (prostaglandin analogues or a combination of prostaglandin analogues and beta-blockers) for regulating the IOP behaviour in POAG patients. Additionally, OPP remains stable after dynamic resistance exercises in both groups. Moderate-intensity dynamic resistance training seems to be a safe training strategy, and thus it should be considered for trainers and eye care specialists working with POAG patients treated with eye drops.

## Supplementary Information

Below is the link to the electronic supplementary material.

Supplementary file1 (XLSX 201 KB)
